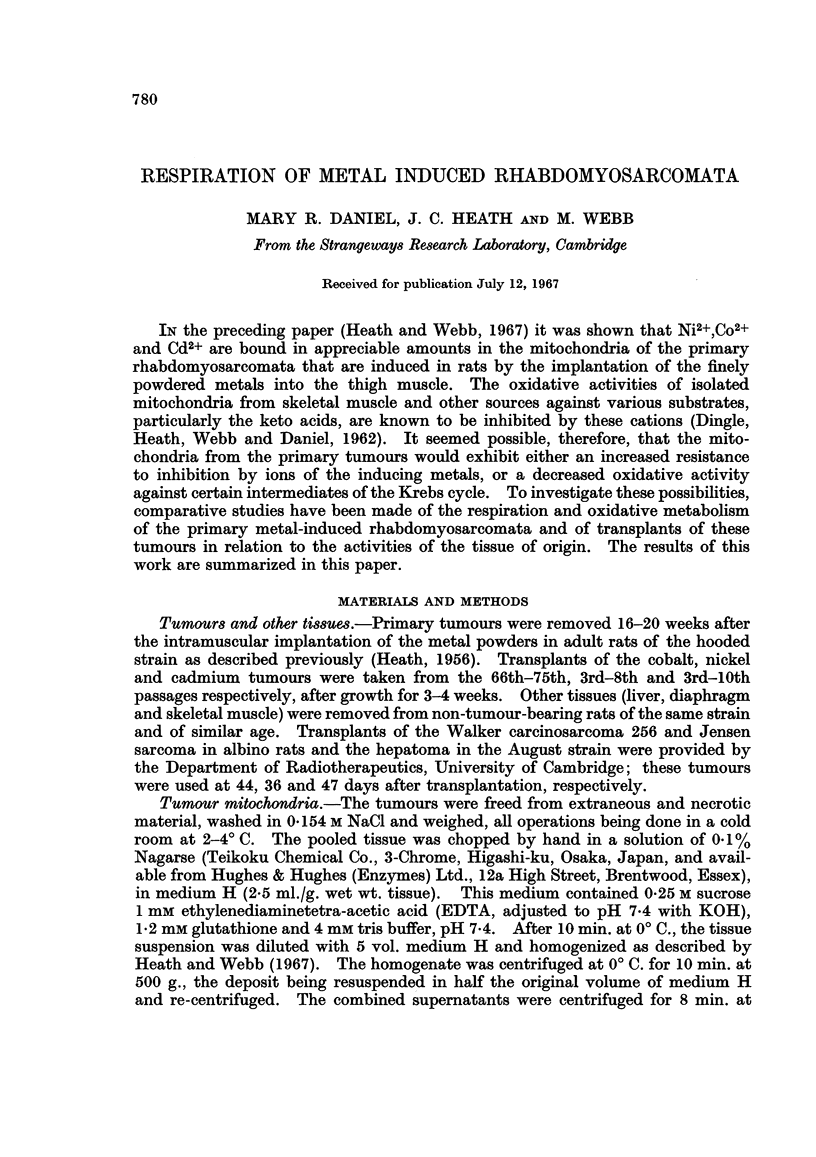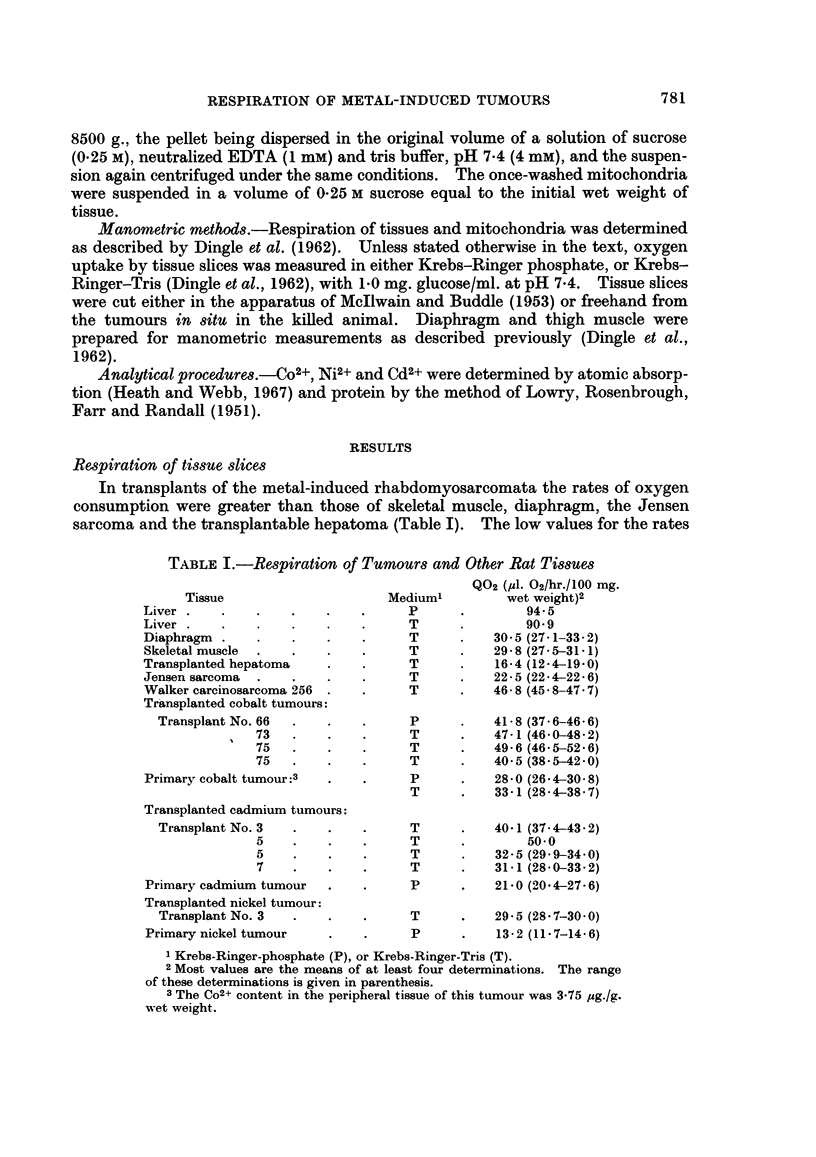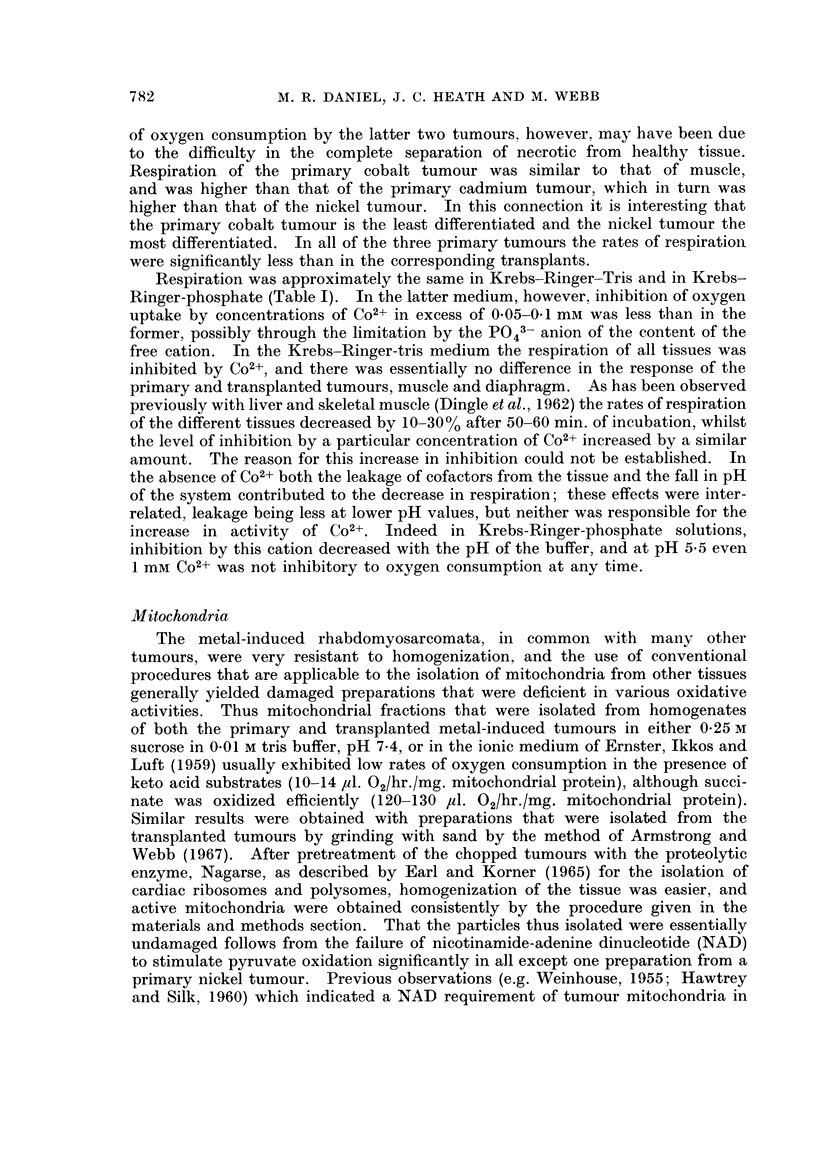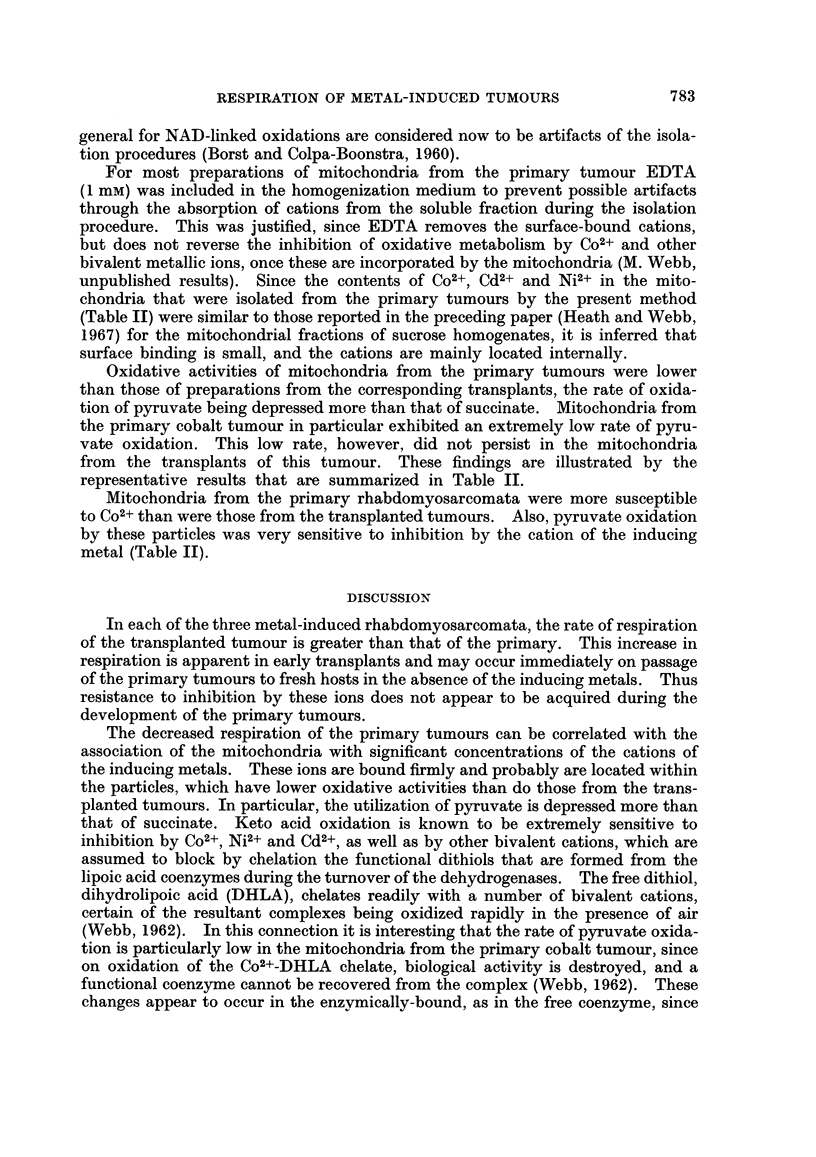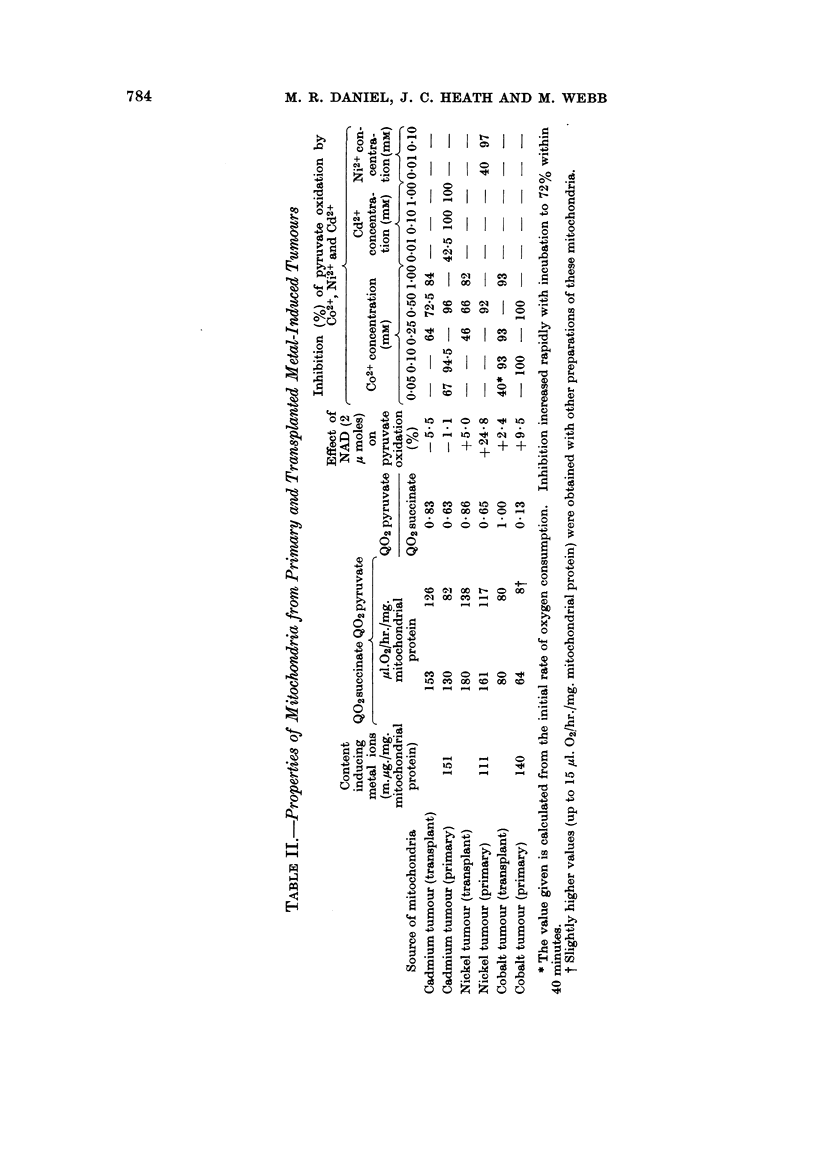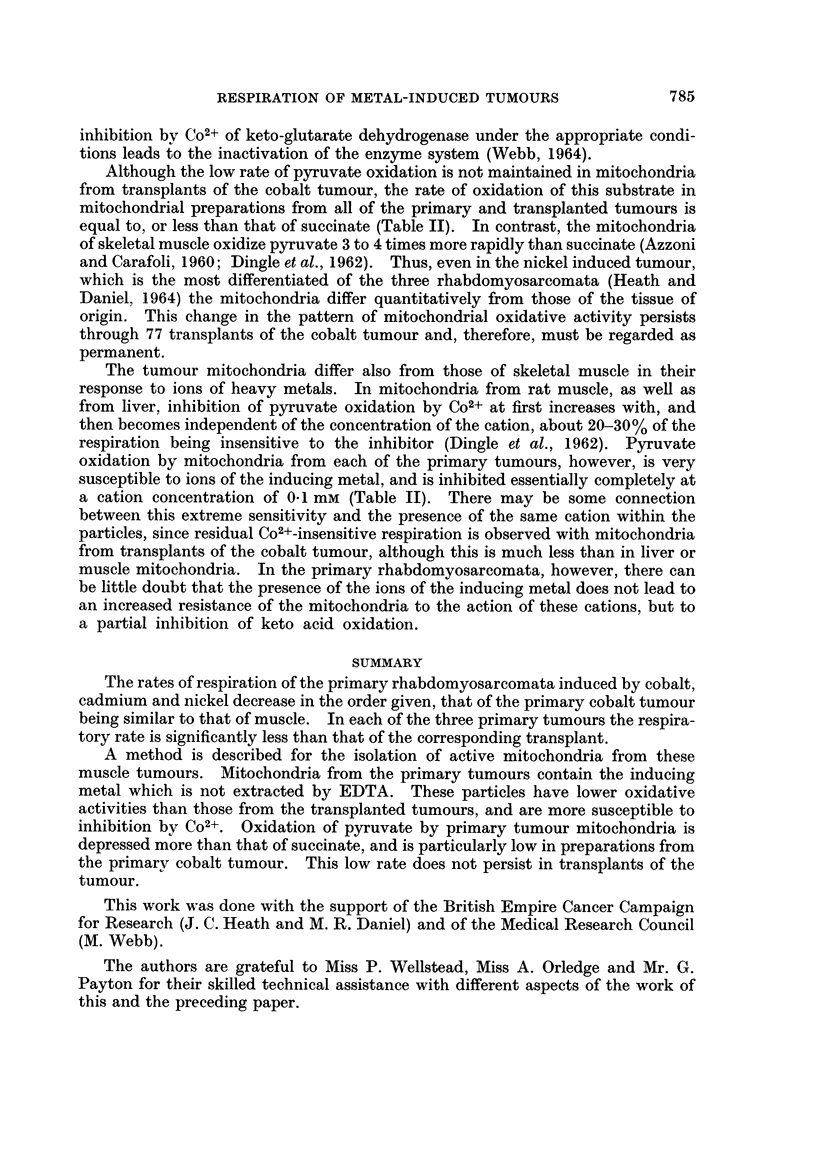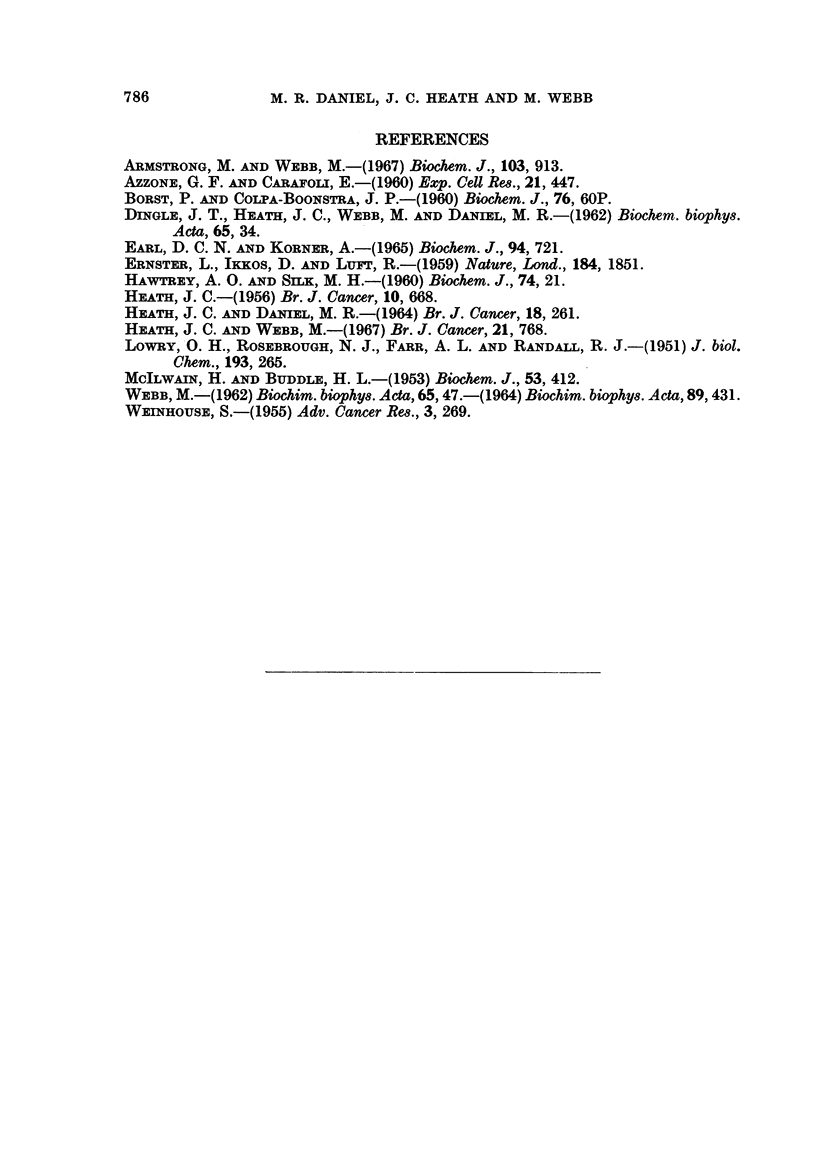# Respiration of metal induced rhabdomyosarcomata.

**DOI:** 10.1038/bjc.1967.91

**Published:** 1967-12

**Authors:** M. R. Daniel, J. C. Heath, M. Webb


### RESPIRATION OF METAL INDUCED RHABDOMYOSARCOMATA

MARY R. DANIEL, J. C. HEATH AND M. WEBB

*From the Strangeways Research Laboratory, Cambridge*

Received for publication July 12, 1967

IN the preceding paper (Heath and Webb, 1967) it was shown that $Ni^{2+}$,$Co^{2+}$ and $Cd^{2+}$ are bound in appreciable amounts in the mitochondria of the primary rhabdomyosarcomata that are induced in rats by the implantation of the finely powdered metals into the thigh muscle. The oxidative activities of isolated mitochondria from skeletal muscle and other sources against various substrates, particularly the keto acids, are known to be inhibited by these cations (Dingle, Heath, Webb and Daniel, 1962). It seemed possible, therefore, that the mitochondria from the primary tumours would exhibit either an increased resistance to inhibition by ions of the inducing metals, or a decreased oxidative activity against certain intermediates of the Krebs cycle. To investigate these possibilities, comparative studies have been made of the respiration and oxidative metabolism of the primary metal-induced rhabdomyosarcomata and of transplants of these tumours in relation to the activities of the tissue of origin. The results of this work are summarized in this paper.

### MATERIALS AND METHODS

*Tumours and other tissues*.—Primary tumours were removed 16–20 weeks after the intramuscular implantation of the metal powders in adult rats of the hooded strain as described previously (Heath, 1956). Transplants of the cobalt, nickel and cadmium tumours were taken from the 66th–75th, 3rd–8th and 3rd–10th passages respectively, after growth for 3–4 weeks. Other tissues (liver, diaphragm and skeletal muscle) were removed from non-tumour-bearing rats of the same strain and of similar age. Transplants of the Walker carcinosarcoma 256 and Jensen sarcoma in albino rats and the hepatoma in the August strain were provided by the Department of Radiotherapeutics, University of Cambridge; these tumours were used at 44, 36 and 47 days after transplantation, respectively.

*Tumour mitochondria*.—The tumours were freed from extraneous and necrotic material, washed in 0·154 M NaCl and weighed, all operations being done in a cold room at 2–4° C. The pooled tissue was chopped by hand in a solution of 0·1% Nagarse (Teikoku Chemical Co., 3-Chrome, Higashi-ku, Osaka, Japan, and available from Hughes & Hughes (Enzymes) Ltd., 12a High Street, Brentwood, Essex), in medium H (2·5 ml./g. wet wt. tissue). This medium contained 0·25 M sucrose 1 mM ethylenediaminetetra-acetic acid (EDTA, adjusted to pH 7·4 with KOH), 1·2 mM glutathione and 4 mM tris buffer, pH 7·4. After 10 min. at 0° C., the tissue suspension was diluted with 5 vol. medium H and homogenized as described by Heath and Webb (1967). The homogenate was centrifuged at 0° C. for 10 min. at 500 g., the deposit being resuspended in half the original volume of medium H and re-centrifuged. The combined supernatants were centrifuged for 8 min. at

8500 g., the pellet being dispersed in the original volume of a solution of sucrose (0·25 M), neutralized EDTA (1 mM) and tris buffer, pH 7·4 (4 mM), and the suspension again centrifuged under the same conditions. The once-washed mitochondria were suspended in a volume of 0·25 M sucrose equal to the initial wet weight of tissue.

*Manometric methods.*—Respiration of tissues and mitochondria was determined as described by Dingle *et al.* (1962). Unless stated otherwise in the text, oxygen uptake by tissue slices was measured in either Krebs–Ringer phosphate, or Krebs–Ringer–Tris (Dingle *et al.*, 1962), with 1·0 mg. glucose/ml. at pH 7·4. Tissue slices were cut either in the apparatus of McIlwain and Buddle (1953) or freehand from the tumours *in situ* in the killed animal. Diaphragm and thigh muscle were prepared for manometric measurements as described previously (Dingle *et al.*, 1962).

*Analytical procedures.*—$Co^{2+}$, $Ni^{2+}$ and $Cd^{2+}$ were determined by atomic absorption (Heath and Webb, 1967) and protein by the method of Lowry, Rosenbrough, Farr and Randall (1951).

### RESULTS

### *Respiration of tissue slices*

In transplants of the metal-induced rhabdomyosarcomata the rates of oxygen consumption were greater than those of skeletal muscle, diaphragm, the Jensen sarcoma and the transplantable hepatoma (Table I). The low values for the rates

TABLE I.—*Respiration of Tumours and Other Rat Tissues*

| Tissue | Medium[1] | $QO_2$ (μl. $O_2$/hr./100 mg. wet weight)[2] |
|---|---|---|
| Liver | P | 94·5 |
| Liver | T | 90·9 |
| Diaphragm | T | 30·5 (27·1–33·2) |
| Skeletal muscle | T | 29·8 (27·5–31·1) |
| Transplanted hepatoma | T | 16·4 (12·4–19·0) |
| Jensen sarcoma | T | 22·5 (22·4–22·6) |
| Walker carcinosarcoma 256 | T | 46·8 (45·8–47·7) |
| Transplanted cobalt tumours: | | |
| Transplant No. 66 | P | 41·8 (37·6–46·6) |
| 73 | T | 47·1 (46·0–48·2) |
| 75 | T | 49·6 (46·5–52·6) |
| 75 | T | 40·5 (38·5–42·0) |
| Primary cobalt tumour:[3] | P | 28·0 (26·4–30·8) |
| | T | 33·1 (28·4–38·7) |
| Transplanted cadmium tumours: | | |
| Transplant No. 3 | T | 40·1 (37·4–43·2) |
| 5 | T | 50·0 |
| 5 | T | 32·5 (29·9–34·0) |
| 7 | T | 31·1 (28·0–33·2) |
| Primary cadmium tumour | P | 21·0 (20·4–27·6) |
| Transplanted nickel tumour: | | |
| Transplant No. 3 | T | 29·5 (28·7–30·0) |
| Primary nickel tumour | P | 13·2 (11·7–14·6) |

[1] Krebs-Ringer-phosphate (P), or Krebs-Ringer-Tris (T).
[2] Most values are the means of at least four determinations. The range of these determinations is given in parenthesis.
[3] The $Co^{2+}$ content in the peripheral tissue of this tumour was 3·75 μg./g. wet weight.

of oxygen consumption by the latter two tumours, however, may have been due to the difficulty in the complete separation of necrotic from healthy tissue. Respiration of the primary cobalt tumour was similar to that of muscle, and was higher than that of the primary cadmium tumour, which in turn was higher than that of the nickel tumour. In this connection it is interesting that the primary cobalt tumour is the least differentiated and the nickel tumour the most differentiated. In all of the three primary tumours the rates of respiration were significantly less than in the corresponding transplants.

Respiration was approximately the same in Krebs–Ringer–Tris and in Krebs–Ringer-phosphate (Table I). In the latter medium, however, inhibition of oxygen uptake by concentrations of $Co^{2+}$ in excess of 0·05–0·1 mM was less than in the former, possibly through the limitation by the $PO_4^{3-}$ anion of the content of the free cation. In the Krebs–Ringer-tris medium the respiration of all tissues was inhibited by $Co^{2+}$, and there was essentially no difference in the response of the primary and transplanted tumours, muscle and diaphragm. As has been observed previously with liver and skeletal muscle (Dingle *et al.*, 1962) the rates of respiration of the different tissues decreased by 10–30% after 50–60 min. of incubation, whilst the level of inhibition by a particular concentration of $Co^{2+}$ increased by a similar amount. The reason for this increase in inhibition could not be established. In the absence of $Co^{2+}$ both the leakage of cofactors from the tissue and the fall in pH of the system contributed to the decrease in respiration; these effects were inter-related, leakage being less at lower pH values, but neither was responsible for the increase in activity of $Co^{2+}$. Indeed in Krebs-Ringer-phosphate solutions, inhibition by this cation decreased with the pH of the buffer, and at pH 5·5 even 1 mM $Co^{2+}$ was not inhibitory to oxygen consumption at any time.

*Mitochondria*

The metal-induced rhabdomyosarcomata, in common with many other tumours, were very resistant to homogenization, and the use of conventional procedures that are applicable to the isolation of mitochondria from other tissues generally yielded damaged preparations that were deficient in various oxidative activities. Thus mitochondrial fractions that were isolated from homogenates of both the primary and transplanted metal-induced tumours in either 0·25 M sucrose in 0·01 M tris buffer, pH 7·4, or in the ionic medium of Ernster, Ikkos and Luft (1959) usually exhibited low rates of oxygen consumption in the presence of keto acid substrates (10–14 $\mu$l. $O_2$/hr./mg. mitochondrial protein), although succinate was oxidized efficiently (120–130 $\mu$l. $O_2$/hr./mg. mitochondrial protein). Similar results were obtained with preparations that were isolated from the transplanted tumours by grinding with sand by the method of Armstrong and Webb (1967). After pretreatment of the chopped tumours with the proteolytic enzyme, Nagarse, as described by Earl and Korner (1965) for the isolation of cardiac ribosomes and polysomes, homogenization of the tissue was easier, and active mitochondria were obtained consistently by the procedure given in the materials and methods section. That the particles thus isolated were essentially undamaged follows from the failure of nicotinamide-adenine dinucleotide (NAD) to stimulate pyruvate oxidation significantly in all except one preparation from a primary nickel tumour. Previous observations (e.g. Weinhouse, 1955; Hawtrey and Silk, 1960) which indicated a NAD requirement of tumour mitochondria in general for NAD-linked oxidations are considered now to be artifacts of the isolation procedures (Borst and Colpa-Boonstra, 1960).

For most preparations of mitochondria from the primary tumour EDTA (1 mM) was included in the homogenization medium to prevent possible artifacts through the absorption of cations from the soluble fraction during the isolation procedure. This was justified, since EDTA removes the surface-bound cations, but does not reverse the inhibition of oxidative metabolism by $Co^{2+}$ and other bivalent metallic ions, once these are incorporated by the mitochondria (M. Webb, unpublished results). Since the contents of $Co^{2+}$, $Cd^{2+}$ and $Ni^{2+}$ in the mitochondria that were isolated from the primary tumours by the present method (Table II) were similar to those reported in the preceding paper (Heath and Webb, 1967) for the mitochondrial fractions of sucrose homogenates, it is inferred that surface binding is small, and the cations are mainly located internally.

Oxidative activities of mitochondria from the primary tumours were lower than those of preparations from the corresponding transplants, the rate of oxidation of pyruvate being depressed more than that of succinate. Mitochondria from the primary cobalt tumour in particular exhibited an extremely low rate of pyruvate oxidation. This low rate, however, did not persist in the mitochondria from the transplants of this tumour. These findings are illustrated by the representative results that are summarized in Table II.

Mitochondria from the primary rhabdomyosarcomata were more susceptible to $Co^{2+}$ than were those from the transplanted tumours. Also, pyruvate oxidation by these particles was very sensitive to inhibition by the cation of the inducing metal (Table II).

### DISCUSSION

In each of the three metal-induced rhabdomyosarcomata, the rate of respiration of the transplanted tumour is greater than that of the primary. This increase in respiration is apparent in early transplants and may occur immediately on passage of the primary tumours to fresh hosts in the absence of the inducing metals. Thus resistance to inhibition by these ions does not appear to be acquired during the development of the primary tumours.

The decreased respiration of the primary tumours can be correlated with the association of the mitochondria with significant concentrations of the cations of the inducing metals. These ions are bound firmly and probably are located within the particles, which have lower oxidative activities than do those from the transplanted tumours. In particular, the utilization of pyruvate is depressed more than that of succinate. Keto acid oxidation is known to be extremely sensitive to inhibition by $Co^{2+}$, $Ni^{2+}$ and $Cd^{2+}$, as well as by other bivalent cations, which are assumed to block by chelation the functional dithiols that are formed from the lipoic acid coenzymes during the turnover of the dehydrogenases. The free dithiol, dihydrolipoic acid (DHLA), chelates readily with a number of bivalent cations, certain of the resultant complexes being oxidized rapidly in the presence of air (Webb, 1962). In this connection it is interesting that the rate of pyruvate oxidation is particularly low in the mitochondria from the primary cobalt tumour, since on oxidation of the $Co^{2+}$-DHLA chelate, biological activity is destroyed, and a functional coenzyme cannot be recovered from the complex (Webb, 1962). These changes appear to occur in the enzymically-bound, as in the free coenzyme, since

TABLE II.—*Properties of Mitochondria from Primary and Transplanted Metal-Induced Tumours*

| Source of mitochondria | Content inducing metal ions (m.$\mu$g./mg. mitochondrial protein) | $QO_2$ succinate | $QO_2$ pyruvate | $QO_2$ pyruvate / $QO_2$ succinate | Effect of NAD (2 $\mu$ moles) on pyruvate oxidation (%) | Inhibition (%) of pyruvate oxidation by $Co^{2+}$, $Ni^{2+}$ and $Cd^{2+}$ | | | | | | | | | |
|---|---|---|---|---|---|---|---|---|---|---|---|---|---|---|---|
| | | $\mu$l.$O_2$/hr./mg. mitochondrial protein | | | | $Co^{2+}$ concentration (mM) | | | | | $Cd^{2+}$ concentration (mM) | | | $Ni^{2+}$ concentration (mM) | |
| | | | | | | 0·05 | 0·10 | 0·25 | 0·50 | 1·00 | 0·01 | 0·10 | 1·00 | 0·01 | 0·10 |
| Cadmium tumour (transplant) | | 153 | 126 | 0·83 | −5·5 | — | — | 64 | 72·5 | 84 | — | — | — | — | — |
| Cadmium tumour (primary) | 151 | 130 | 82 | 0·63 | −1·1 | 67 | 94·5 | — | 96 | — | 42·5 | 100 | 100 | — | — |
| Nickel tumour (transplant) | | 180 | 138 | 0·86 | +5·0 | — | — | 46 | 66 | 82 | — | — | — | — | — |
| Nickel tumour (primary) | 111 | 161 | 117 | 0·65 | +24·8 | — | — | — | 92 | — | — | — | — | 40 | 97 |
| Cobalt tumour (transplant) | | 80 | 80 | 1·00 | +2·4 | 40* | 93 | 93 | — | 93 | — | — | — | — | — |
| Cobalt tumour (primary) | 140 | 64 | 8† | 0·13 | +9·5 | — | 100 | — | 100 | — | — | — | — | — | — |

* The value given is calculated from the initial rate of oxygen consumption. Inhibition increased rapidly with incubation to 72% within 40 minutes.

† Slightly higher values (up to 15 $\mu$l. $O_2$/hr./mg. mitochondrial protein) were obtained with other preparations of these mitochondria.

inhibition by $Co^{2+}$ of keto-glutarate dehydrogenase under the appropriate conditions leads to the inactivation of the enzyme system (Webb, 1964).

Although the low rate of pyruvate oxidation is not maintained in mitochondria from transplants of the cobalt tumour, the rate of oxidation of this substrate in mitochondrial preparations from all of the primary and transplanted tumours is equal to, or less than that of succinate (Table II). In contrast, the mitochondria of skeletal muscle oxidize pyruvate 3 to 4 times more rapidly than succinate (Azzoni and Carafoli, 1960; Dingle *et al.*, 1962). Thus, even in the nickel induced tumour, which is the most differentiated of the three rhabdomyosarcomata (Heath and Daniel, 1964) the mitochondria differ quantitatively from those of the tissue of origin. This change in the pattern of mitochondrial oxidative activity persists through 77 transplants of the cobalt tumour and, therefore, must be regarded as permanent.

The tumour mitochondria differ also from those of skeletal muscle in their response to ions of heavy metals. In mitochondria from rat muscle, as well as from liver, inhibition of pyruvate oxidation by $Co^{2+}$ at first increases with, and then becomes independent of the concentration of the cation, about 20–30% of the respiration being insensitive to the inhibitor (Dingle *et al.*, 1962). Pyruvate oxidation by mitochondria from each of the primary tumours, however, is very susceptible to ions of the inducing metal, and is inhibited essentially completely at a cation concentration of 0·1 mM (Table II). There may be some connection between this extreme sensitivity and the presence of the same cation within the particles, since residual $Co^{2+}$-insensitive respiration is observed with mitochondria from transplants of the cobalt tumour, although this is much less than in liver or muscle mitochondria. In the primary rhabdomyosarcomata, however, there can be little doubt that the presence of the ions of the inducing metal does not lead to an increased resistance of the mitochondria to the action of these cations, but to a partial inhibition of keto acid oxidation.

### SUMMARY

The rates of respiration of the primary rhabdomyosarcomata induced by cobalt, cadmium and nickel decrease in the order given, that of the primary cobalt tumour being similar to that of muscle. In each of the three primary tumours the respiratory rate is significantly less than that of the corresponding transplant.

A method is described for the isolation of active mitochondria from these muscle tumours. Mitochondria from the primary tumours contain the inducing metal which is not extracted by EDTA. These particles have lower oxidative activities than those from the transplanted tumours, and are more susceptible to inhibition by $Co^{2+}$. Oxidation of pyruvate by primary tumour mitochondria is depressed more than that of succinate, and is particularly low in preparations from the primary cobalt tumour. This low rate does not persist in transplants of the tumour.

This work was done with the support of the British Empire Cancer Campaign for Research (J. C. Heath and M. R. Daniel) and of the Medical Research Council (M. Webb).

The authors are grateful to Miss P. Wellstead, Miss A. Orledge and Mr. G. Payton for their skilled technical assistance with different aspects of the work of this and the preceding paper.

### REFERENCES

Armstrong, M. and Webb, M.—(1967) *Biochem. J.*, **103**, 913.
Azzone, G. F. and Carafoli, E.—(1960) *Exp. Cell Res.*, **21**, 447.
Borst, P. and Colpa-Boonstra, J. P.—(1960) *Biochem. J.*, **76**, 60P.
Dingle, J. T., Heath, J. C., Webb, M. and Daniel, M. R.—(1962) *Biochem. biophys. Acta*, **65**, 34.
Earl, D. C. N. and Korner, A.—(1965) *Biochem. J.*, **94**, 721.
Ernster, L., Ikkos, D. and Luft, R.—(1959) *Nature, Lond.*, **184**, 1851.
Hawtrey, A. O. and Silk, M. H.—(1960) *Biochem. J.*, **74**, 21.
Heath, J. C.—(1956) *Br. J. Cancer*, **10**, 668.
Heath, J. C. and Daniel, M. R.—(1964) *Br. J. Cancer*, **18**, 261.
Heath, J. C. and Webb, M.—(1967) *Br. J. Cancer*, **21**, 768.
Lowry, O. H., Rosebrough, N. J., Farr, A. L. and Randall, R. J.—(1951) *J. biol. Chem.*, **193**, 265.
McIlwain, H. and Buddle, H. L.—(1953) *Biochem. J.*, **53**, 412.
Webb, M.—(1962) *Biochim. biophys. Acta*, **65**, 47.—(1964) *Biochim. biophys. Acta*, **89**, 431.
Weinhouse, S.—(1955) *Adv. Cancer Res.*, **3**, 269.